# Changes in prevalence of psychotropic drug use and alcohol consumption among the elderly in Germany: results of two National Health Interview and Examination Surveys 1997-99 and 2008-11

**DOI:** 10.1186/s12888-017-1254-x

**Published:** 2017-03-09

**Authors:** Ingrid-Katharina Wolf, Yong Du, Hildtraud Knopf

**Affiliations:** Department of Epidemiology and Health Monitoring, Robert Koch Institute, General-Pape-Str. 64-66, D-12101 Berlin, Germany

**Keywords:** Changes, Psychotropic drugs, Alcohol, National health surveys, Older adults, Germany

## Abstract

**Background:**

Psychotropic drug use and alcohol consumption among older adults need to be monitored over time as their use or combined use bears risks of harms. Representative data on changes in prevalence, patterns and co-relates of substance use are lacking in Germany.

**Methods:**

Participants were older adults (60–79 years) from two German National Health Surveys: 1997–99 (GNHIES98, *N* = 1,606) and 2008-11 (DEGS1, *N* = 2,501). Included were drugs acting on the nervous system used during the last 7 days. Alcohol consumption was measured by frequency (daily drinking) and quantity (risky drinking: ≥20/10 g/day alcohol for men/women). Changes in prevalence adjusted for potential socio-economic and health-related confounders were calculated by logistic regression models approximated by the SAS LSMEANS statement.

**Results:**

The prevalence of overall psychotropic drug use (20.5% vs. 21.4%) remained constant between the two surveys. Significant changes were observed in the use of some psychotropics (all GNHIES98 vs. DEGS1): Synthetic antidepressants (3.9% vs. 6.9%), St. John’s wort (2.9% vs. 1.1%), benzodiazepines (3.7% vs. 2.5%), benzodiazepine related drugs (0.2% vs. 0.8%), narcotic analgesics (3.0% vs. 4.1%), anti-dementia drugs (2.2% vs. 4.2%) and anti-epileptics (1.0% vs. 2.3%). Significant changes were also observed in long-term use of synthetic anti-depressants (3.2% vs. 5.9%), St. John’s wort (2.0% vs. 0.6%) and opioid analgesics (1.0% vs. 2.2%). Further, we found significant changes in benzodiazepines use (3.3% vs. 1.4%) among men, opioids use (2.9% vs. 7.3%) among people with a lower social status, and overall psychotropics (26.8% vs. 32.5%) as well as opioids use (4.4% vs. 8.1%) among those with a worse health status. Moderate alcohol consumption increased significantly (58.0% vs. 66.9%). Risky drinking remained unchanged (16.6% vs. 17.0%). In spite of significant increases in daily alcohol drinking (13.2% vs. 18.4%) psychotropic drug use combined with daily drinking remained unchanged (1.8% vs. 2.7%).

**Conclusions:**

Although prevalence of overall psychotropic drug use remained stable, changes in the use of some psychotropic drug groups and alcohol consumption patterns have been observed. Further studies are required to investigate resulting health consequences and public health relevance of those outcomes.

## Background

The prevalence of psychotropic drug and alcohol-interactive medicine use rises sharply with older age [1]. An age-related increase of multi-morbidity and polypharmacy bears higher risks of adverse drug reactions and a reduced drug metabolism in older people aggravates those risks [2]. Potential adverse health outcomes of psychotropic drug use such as falls, strokes or mortality are described in literature [3–5].

The long-term use of some psychotropic drugs, such as opioids and benzodiazepines, can lead to dependency and addiction. With the exception of some medically necessary indications those drugs should be used only for a short period [6, 7].

Psychotropic drugs of phytoceutical origin are also of interest as in Germany the use of alternative medicine has a long tradition and is very common [8].

Alcohol has a psychotropic effect, an addictive potential, and bears health risks for the elderly. According to the Organisation for Economic Co-operation and Development (OECD) alcohol consumption in Germany is with 11 l per capita higher than the average European consumption level of 10 l. Additionally, among adults about every sixth woman and about every third man consumes alcohol above tolerable upper levels for healthy adults of 10-12 g/20-24/day for women/men [9, 10]. The combined use of alcohol and psychotropic medicine may aggravate potential risks of both substances [5].

Drug use changes over time as new drugs are introduced into the market and indications for existing drugs might be changed. Also, changes in drug use might be influenced by changes in guidelines for the use of drugs. Transparency on the use of medications and on changes in the use over time is a prerequisite for an informed Public Health Policy.

Prescription data from the statutory health insurance system can provide information on changes in prescription over time but cannot reproduce the real consumption of medicines. Patients might not take all tablets of a package; self-medication and private prescriptions are not recorded by prescription data. Further, health insurance data cannot be linked with a variety of population characteristics, such as socio-economic and health behavior factors (e.g. alcohol consumption).

In Germany, population based data from two comparable surveys - the German National Health Interview and Examination Survey 1997-99 (GNHIES98) and the German Health Interview and Examination Survey for Adults 2008-11 (DEGS1) – with data on the use of psychotropic drugs and alcohol in the older population (60–79 years) are available. A cross-sectional analysis of psychotropic drug use, alcohol consumption and the combined use of both substances based on DEGS1 data has been conducted by the authors recently [11].

Representative population based information on changes over time in psychotropic substance use among the elderly and information on changes in the use of psychotropics according to socio-economic and health-behavior factors are lacking in Germany.

In the present work we analyzed changes in psychotropic drug use, alcohol consumption, and the concomitant use of psychotropic drugs and alcohol between 1997-99 and 2008-11. Additionally, we examined changes in long-term use of selected subgroups of psychotropic drugs. Further, by analyzing changes over time according to socio-economic and health-related factors we identified particularly vulnerable groups among older adults in Germany.

## Methods

### Study population

Analyses conducted in this study were based on data from two nationally representative surveys among non-institutionalized adults: GNHIES98 (1997-99) and DEGS1 (2008-11). Both surveys were conducted by the Robert Koch Institute (RKI) and followed largely the same sampling strategy and study protocol. This has been described in detail previously [12, 13]. Briefly, both surveys recruited participants via a two-stage sampling design. In the first stage a nationwide selection of communities which were representative of location, size, and structure of German communities was conducted (GNHIES98: 120 and DEGS1: 180 communities). In a second stage a representative sample of the 18–79 year old population was drawn from local population registries of the chosen communities. All participants from GNHIES98 (*N* = 7.124) were invited to re-participate in DEGS1. 3,959 women and men (response rate 62%) followed this invitation. In order to achieve a nationally representative sample 4,193 first time participants were also included in DEGS1 (response rate 42%), amounting to a total study population of *N* = 8.152. Non-responder analyses in both surveys displayed no significant differences between participants and non-participants regarding demographic factors [14, 15].

The present analyses focused on men and women aged 60–79 years (GNHIES98: *N* = 1,615 and DEGS1: *N* = 2,508). Among those only participants who completed the interview on medication use were included (GNHIES98: *N* = 1,606 and DEGS1: *N* = 2,501).

### Data collection

Both surveys employed identical data collection methods including self-administered questionnaires, medical examinations, physical measurements, laboratory tests, standardized physician administered computer assisted personal interviews (CAPI), and standardized personal interviews on medicine use [12]. Information on socio-demographic factors (e.g. age, sex, region of residence, household size, income, profession, and education) as well as health-related factors (e.g. self-perceived health status, presence of an officially certified disability, and alcohol consumption) was collected via standardized, self-administered questionnaires [12].

### Identification of psychotropic drug use and alcohol consumption

In the invitation letter participants were asked to bring along original packages of the drugs they had taken during the preceding 7 days, including Over-The Counter (OTC) products and dietary supplements. Detailed information on each preparation was recorded by trained health professionals. For all drugs the brand name, indication, frequency (taken when needed or regular), and duration of use were recorded. The preparations were classified by the Anatomical Therapeutic Chemical (ATC) codes. In the present analyses we included drugs belonging to the nervous system class (ATC code N00). The following drug groups were included: Narcotic analgesics (N02A) and aspirin if combined with caffeine (N02BA71), all anti-epileptics (N03), all anti-parkinson drugs (N04), all psycholeptics (N05) with hypnotics and sedatives (N05C), benzodiazepines (N05BA, N05CD, N03AE01)) and benzodiazepine-related drugs (N05CF), all psychoanaleptics (N06) with anti-depressants (N06A) and anti-dementia drugs (N06D), all other nervous system drugs (N07) as well as opiates used as antitussives (ATC code R05DA), and psychotropic drugs with herbal active ingredients (ATC code N05CP or N06AP or N06DP). Excluded were: Other analgesics and antipyretics such as aspirin and paracetamol (ATC code N02B), local anesthetics (ATC code N01B), homeopathic drugs of the ATC class N00, and drugs with indistinctive active ingredients. Concerning frequency and duration of drug use we differentiated between drug use “if needed”, “regular, < 3 months” and “regular, ≥ 3 months”. The cut-off point of ≥ 3 months for long-term use was chosen as some psychotropic drugs can lead to addiction. Guidelines on the use of opioids in non-cancer pain recommend an application time of no longer than 3 months [6]. The recommendations for limitations in the use of benzodiazepines are even shorter [7, 16]. Anti-depressants have to be taken for a longer period in order to have a therapeutic effect, are usually not taken “only if needed”, and their dependency potential is much lower compared to opioids or benzodiazepines [17]. Changes in the prevalence of psychotropic drug use were analyzed for drugs of the above listed ATC classes. Additionally, changes in long-term use were analyzed for the most frequently used drug groups antidepressants, as well as for benzodiazepines and opioids as their long-term use harbors a potential for dependency and addiction.

Alcohol consumption during the preceding 12 months was recorded via a standardized food-frequency questionnaire and was classified according to frequency as well as quantity. Within the classification according to frequency we differentiated between alcohol consumption “at least once a week” (weekly) and “at least once a day” (daily). For the classification according to quantity the mean amount of pure alcohol consumed in grams per day was calculated according to methods previously described [18]. Alcohol consumption according to quantity was classified as “moderate drinking” and “risky drinking”. The definition of risky drinking varies greatly internationally [19]. In Germany for healthy younger people a limit of ≥10–12 g/day for women and ≥20–24 g/day for men is assumed [18, 19]. As older people could be at risk when consuming much smaller amounts of alcohol, and as there are no internationally agreed limits for risky alcohol consumption among the elderly, we adopted the lower limits of >0 to <10 g for women and >0 to <20 g for men to classify “moderate drinking” and ≥10 g/day for women and ≥20 g/day for men to classify “risky drinking”. For measurement of the combined use of psychotropic drugs and alcohol we chose the categories “daily drinking“and “daily risky drinking” as this offers the strongest probability that both substances are taken simultaneously.

### Co-variables

As co-variables we included sex, age group (60–69 and 70–79 years), social status according to a total score of a composite social status index [20] (lower, middle, upper), living alone (only one person living in a household), and urbanity (rural: <5000; small city: 5000 - <20.000; medium sized city: 20.000 - <100.000; large city: 100.000 and more residents). To determine region of residence we divided Germany into three commonly described geographical areas, each including several federal states: Northern Germany (Berlin, Brandenburg, Bremen, Hamburg, Lower Saxony, Mecklenburg-West Pomerania, and Schleswig-Holstein); Central Germany (Hesse, North Rhine-Westphalia, Saxony, Saxony-Anhalt, and Thuringia) and Southern Germany (Baden-Württemberg, Bavaria, Rhineland-Palatinate and Saarland). The following health-related co-variables were included: Self-assessed health status dichotomized as “better” (very good or good) and “worse” (moderate, bad, and very bad), having an officially certified disability (yes, no) and exposure to polypharmacy (five or more different products, prescribed and/or OTC within the past seven days, excluding psychotropic drugs).

### Statistical analyses

To generate population representative prevalence rates of psychotropic substance use in trend analyses a weighting factor was introduced. This served the correction of any deviations in the samples from population structure regarding age, sex, region of residence, municipality size, nationality, and educational level in comparison to the German population of the 31th December 2010 [15]. Re-participation probability of GNHIES98 participants was also included for calculation of the weighting factor. Characteristics of the population were analyzed via descriptive statistics. For the prevalence of psychotropic drug use and alcohol consumption, we calculated the absolute changes in percentage points (%) and 95% Confidence Intervals (95% CIs) between surveys, both unadjusted and adjusted for co-variables. The adjusted changes in percentage points were derived from the predictive margins calculated from a logistic regression model [21, 22]. First-order interactions between the survey year and the co-variates were tested and included in the model if *p* < 0.10. The predictive margins were calculated as the adjusted probabilities predicted by the model, averaged over all subjects in the model and assuming that the co-variable distribution in GNHIES98 and DEGS1 was identical. The standard errors and correlation of the predictive margins were approximated by the SAS LSMEANS statement and used in the calculation of the 95% CIs for the adjusted changes in percentage points. SAS 9.4 survey procedures for complex samples (SAS Institute Inc., Cary, NC) or SPSS complex samples module were used for statistical analyses. A probability level for statistically significant group differences was considered at *p* < 0.05 based on two-sided tests.

## Results

### Characteristics of study-populations

There were no significant differences between the GNHIES98 and the DEGS1 population concerning socio-demographics (age, sex, urbanity, regions of residence, and social status) as well as the proportion of people living alone or having an officially certified disability (Table 1). Significant differences between the two survey-populations were found for “self-assessed health status” and “polypharmacy”. More people rated their health as better in 2008–11 than in 1997–99. Contrarily, more people in 2008–11 used polypharmacy (Table 1).

**Table 1:** Descriptive characteristics of study populations aged 60-79 years. National Health Interview and Examination Surveys GNHIES98 (1997–99) and DEGS1 (2008–11)

	GNHIES98^a^ (*N* = 1606)				DEGS1^a^ (*N* = 2501)				*P* value
	*n*	%	95% C		*n*	%	95% CI		
Sex									
Men	727	47.0	44.7	49.3	1227	46.9	44.5	49.3	.962
Women	879	53.0	50.7	55.3	1274	53.1	50.7	55.5	
Age groups									
60–69 years	1031	52.5	49.9	55.1	1393	52.6	50.6	54.5	.970
70–79 years	575	47.5	44.9	50.1	1108	47.4	45.5	49.4	
Living alone									
Yes	355	24.2	21.0	27.7	506	21.3	19.2	23.6	.084
No	1174	75.8	72.3	79.0	1983	78.7	76.4	80.8	
Urbanity^b^									
Rural	382	19.9	13.2	29.0	428	16.3	11.0	23.6	.113
Small city	333	18.9	12.4	27.8	586	25.6	19.0	33.6	
Medium sized city	406	28.1	20.0	37.9	733	27.8	21.2	35.6	
Large city	485	33.1	24.1	43.4	754	30.2	23.3	38.2	
Region of residence^c^									
Northern Germany	375	26.0	18.0	35.9	641	25.6	19.1	33.5	.979
Central Germany	781	40.7	31.5	50.7	1137	41.4	33.6	49.6	
Southern Germany	450	33.3	24.3	43.7	723	33.0	25.7	41.2	
Social status									
Lower	374	26.1	22.8	29.6	436	24.1	21.2	27.3	.393
Middle	935	59.1	55.8	62.4	1489	59.3	56.3	62.3	
Upper	233	14.8	12.1	17.9	562	16.5	14.6	18.7	
Officially certified disability									
Yes	371	26.0	23.3	29.0	647	29.1	26.4	31.9	.079
No	1168	74.0	71.0	76.7	1797	70.9	68.1	73.6	
Self-assed health status									
Better	781	47.8	44.4	51.1	1498	58.1	55.5	60.7	**<.0001**
Worse	825	52.2	48.9	55.6	985	41.9	39.3	44.5	
Polypharmacy^d^									
Yes	466	30.0	27.1	33.0	993	38.8	36.5	41.2	**<.0001**
No	1140	70.0	67.0	72.9	1508	61.2	58.8	63.5	

^a^ Standardized to the population of 1.12.2010

^b^ Urbanity: Rural (<5000 residents), small city (5000 - <20.000), medium sized city (20.000 - <100.000), large city (100.000 and more residents)

^c^ Regions: Northern Germany (federal states: Berlin, Brandenburg, Bremen, Hamburg, Lower Saxony, Mecklenburg-Vorpommern, Schleswig-Holstein), Central Germany (Hesse, North Rhine-Westphalia, Saxony, Saxony-Anhalt, Thuringia), Southern Germany (Baden-Württemberg, Bavaria, Rhineland-Palatinate, Saarland)

^d^ Polypharmacy: Use of five or more different prescribed and OTC drugs (excluding psychotropics) in the last seven days

Missing values: Living alone (GNHIES98 *n* = 77, DEGS1 *n* = 12), social status (64, 14), officially certified disability (67, 57), self-assed health status (0, 18)

Figures in bold denote statistical significance

### Changes in psychotropic drug use, alcohol consumption and combined use of both substances

Table 2 depicts the changes in psychotropic drug use and alcohol consumption from 1997–99 to 2008–11 adjusted for sex, age group, region of residence, community size, social status, polypharmacy, living alone and having a recognized disability. In the models self-assessed health status was not included as an adjusting factor as it correlates to a large extend with polypharmacy. Repeated analyses with self-assessed health status replacing polypharmacy in models showed no significant changes.

**Table 2:** Changes in the prevalence of psychotropic drug use and alcohol consumption among adults aged 60–79 years in Germany. National Health Interview and Examination Surveys GNHIES98 (1997–99) and DEGS1 (2008–11)

	GNHIES98^a^				DEGS1^a^				Change in % (GNHIES98-DEGS1)							
	*n*	%	95% CI		*n*	%	95% CI		Unadjusted^a^	95% CI		*p* value	Adjusted^a,b^	95% CI		*p* value
All psychotropic drugs (*synthetics & phytoceuticals)*	313	20.5	18.2	22.9	518	21.4	19.3	23.7	1.0	-2.2	4.1	.554	-0.3	-5.4	4.8	0.897
All phytoceuticals	107	6.7	5.4	8.3	158	6.5	5.4	7.8	-0.2	-1.9	1.5	.827	-0.1	-2.3	2.2	0.956
All synthetics	223	14.6	12.6	16.9	404	16.9	14.9	19.1	2.3	-0.8	5.3	.145	1.5	-4.3	7.2	0.622
Weekly alcohol use (at least once a week)	739	48.8	45.5	52.2	1295	51.0	48.1	53.9	2.1	-2.0	6.2	.305	4.0	-3.8	11.8	0.319
Daily alcohol use (at least once a day)	185	13.2	11.1	15.5	468	18.4	16.3	20.7	**5.2**	2.4	8.1	**.001**	**9.6**	5.7	13.6	**<.001**
Moderate drinking^c^	919	58.0	54.6	61.3	1659	66.9	64.1	69.5	**8.9**	4.9	13.0	**<.001**	**8.9**	4.6	13.1	**<.001**
Risky drinking^d^	240	16.6	14.2	19.4	459	17.0	14.9	19.2	0.3	-2.7	3.3	.836	0.4	-2.0	2.9	0.724
Psychotropic drugs + daily drinking	25	1.8	1.2	2.7	76	2.7	2.0	3.7	0.9	-0.2	2.1	.122	1.3	-0.3	2.8	0.105
Psychotropic drugs + daily risky drinking	20	1.5	.9	2.4	59	2.1	1.5	2.9	0.6	-0.4	1.6	.274	0.7	-0.5	1.9	0.258

^a^ Standardized to the population of 31.12.2010

^b^ Adjusted for sex, age group, region, community size, social status, polypharmacy, living alone, recognized disability

^c^ Moderate drinking: average daily consumption of alcohol between >0 and <10 g for women, and between >0 and < 20 g for men

^d^ Risky drinking: average daily consumption of alcohol ≥10 g for women, and ≥20 g for men

Figures in bold denote statistical significance

After adjustments no changes over time in the use of overall synthetic or overall phytoceutical psychotropic drugs were found. In all categories of alcohol consumption we observed rising prevalence rates, but they were only significant for daily (adjusted change: +9.6%, *p*- < .0001) and moderate drinking (adjusted change: +8.9% *p*- < .0001). Combined use of psychotropic drugs and daily or daily risky alcohol consumption remained unchanged (Table 2).

### Changes in the use of specific psychotropic drug groups

Table 3 presents the changes in the use of specific groups and subgroups of psychotropic drugs between the surveys of 1997–99 and 2008–11. Prevalence of overall anti-depressants use (6.4% vs. 7.9%) has not changed but we found significant changes in the following subgroups: The use of the phytoceutical antidepressant St. John’s wort declined (adjusted change: -5.0%, *p* = 0.001) and the use of synthetical antidepressants increased (adjusted change: +6.0%, *p* = 0.001). Among synthetical antidepressants we observed a significant rise in the use of selective serotonin reuptake inhibitors (SSRIs) (adjusted change: +4.1%, *p* = <0.001). Prevalence in the use of hypnotics & sedatives (3.6% vs. 3.3%) or overall benzodiazepines & benzodiazepine-related drugs (Z-drugs) (3.9% vs. 2.4%) remained unchanged. However, looking at benzodiazepine and Z-drug use separately, we found a significant decline in the use of benzodiazepines (adjusted change: -3.4%, *p* = 0.024) and a significant rise in the use of Z-drugs (adjusted change: +1.3%, *p* = < 0.001). Further, we observed significant rises in the use of overall anti-dementia drugs (adjusted change: +3.2%, *p* = 0.001) - especially Ginkgo biloba (adjusted change: +2.4%, *p* = 0.001) -, narcotic analgesics (N02A) (adjusted change: +3.9%, *p* = 0.026), and anti-epileptics (adjusted change: +3.6%, *p* = <0.001). There were no significant changes in the use of anti-parkinson drugs (1.1% vs. 1.2%) and anxiolytics (2.4% vs. 2.2%) within the observation period (Table 3).

**Table 3:** Changes in the prevalence of the use of specific psychotropic drug groups among adults aged 60-79 years in Germany. National Health Interview and Examination Surveys GNHIES98 (1997–99) and DEGS1 (2008–11)

	GNHIES98^a^				DEGS1^a^				Change in % (GNHIES98-DEGS1)							
	*n*	%	95% CI		*n*	%	95% CI		Unadjusted^a^	95% CI		*p* value	Adjusted^a,b^	95% CI		*p* value
1. All anti-depressants (St. John’s wort and all synthetical antidepressants)	96	6.4	5.2	7.9	173	7.9	6.5	9.5	1.4	-0.6	3.4	.157	1.7	-1.8	5.2	0.343
1.1 St. John’s wort (N06AP)	47	2.9	2.1	3.9	27	1.1	0.8	1.7	-1.7	-2.7	-0.8	**<.001**	-5.0	-7.9	-2.0	**0.001**
1.2 All synthetical antidepressants	54	3.9	2.9	5.2	151	6.9	5.5	8.5	3.0	1.2	4.8	**.001**	6.0	2.5	9.4	**0.001**
1.2.1. NSMRIs^c^ (N06AA)	45	3.6	2.6	4.8	80	3.8	2.8	5.2	0.3	-1.3	1.8	.741	0.4	-2.7	3.5	0.806
1.2.2. SSRIs^d^ (N06AB)	3	0.3	0.1	1.0	47	2.0	1.4	2.7	1.7	1.0	2.4	.**001**	4.1	2.4	5.8	**<.0001**
2. All hypnotics & sedatives (synth., antihistamines and phytoceuticals)	62	3.6	2.8	4.8	98	3.3	2.6	4.3	-0.3	-1.6	1.0	.644	-1.3	-3.5	0.9	0.234
2.1 All synth. (N05C) and antihistamines (N05CM)	31	1.6	1.1	2.4	52	1.6	1.1	2.2	0.0	-0.9	0.8	.921	-0.1	-1.7	1.4	0.861
2.1.1. Hypnotics & sedatives (N05C)	31	1.6	1.1	2.4	42	1.3	0.9	1.9	-0.3	-1.1	0.5	.430	-0.6	-2.2	0.9	0.412
2.2. All phytoceuticals	32	2.1	1.4	3.0	51	1.9	1.3	2.6	-0.2	-1.2	0.8	.703	-0.6	-1.9	0.6	0.307
2.2.1. Valerian (N05CP)	28	1.8	1.2	2.7	42	1.5	1.0	2.1	-0.3	-1.2	0.6	.453	-0.5	-1.7	0.7	0.408
3. Benzodiazepines and benzodiazepine-related drugs	64	3.9	2.9	5.2	84	3.3	2.4	4.4	-0.6	-2.1	0.8	.389	-2.0	-5.0	0.9	0.176
3.1. Benzodiazepines (N05BA, N05CD, N03AE01)	57	3.7	2.8	5.0	57	2.5	1.8	3.5	-1.2	-2.6	0.2	.081	-3.4	-6.3	-0.4	**0.024**
3.2. Benzodiazepine-related drugs (Z-drugs) (N05CF)	8	0.2	0.1	0.5	28	0.8	0.5	1.3	0.6	0.2	1.0	**.003**	1.3	0.6	2.1	**<0.001**
4. All anti-dementia drugs incl. ginkgo biloba	34	2.2	1.4	3.4	97	4.2	3.3	5.4	2.0	0.7	3.3	**.005**	3.2	1.3	5.1	**0.001**
4.1. Ginkgo biloba (N06DP01)	34	2.2	1.4	3.4	88	3.8	2.9	4.8	1.6	0.3	2.8	**.022**	2.4	1.0	3.8	**0.001**
5. Narcotic analgesics (N02A)	39	3.0	2.1	4.2	96	4.1	3.2	5.3	1.2	-0.3	2.6	.121	3.9	0.5	7.3	**0.026**
6. Anti-epileptics (N03)	14	1.0	0.5	1.7	63	2.3	1.7	3.3	1.4	0.4	2.3	**.008**	3.6	1.7	5.5	**<0.001**
7. Antiparkinson drugs (N04)	19	1.1	.7	1.8	32	1.2	0.7	2.0	0.1	-0.7	0.9	.806	-0.1	-1.4	1.3	0.934
8. Anxiolytics (N05B)	34	2.4	1.6	3.5	48	2.2	1.5	3.1	-0.2	-1.4	1.0	.751	-1.2	-3.2	0.9	0.268

^a^standardized to the population of 31.12.2010

^b^ adjusted for sex, age group, region, community size, social status, polypharmacy, living alone, recognized disability

^c^Non-Selective Monoamine Reuptake Inhibitors

^d^Selective Serotonin Reuptake Inhibitors

Figures in bold denote statistical significance

### Changes in long-term use of psychotropic drugs

**Fig. 1:**
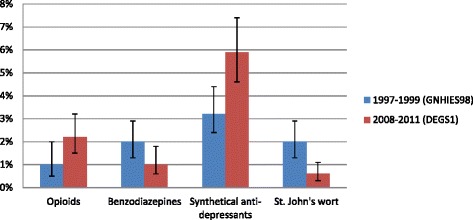
Prevalence of long-term psychotropic drug use among adults aged 60–79 years in Germany. National Health Interview and Examination Surveys GNHIES98 (1997–99) and DEGS1 (2008–11)

Figure 1 depicts changes in prevalence of long-term use for some selected psychotropic drugs between the surveys of 1997–99 and 2008–11. Long-term use of opioid analgesics (1.0% vs. 2.2%, *p* = 0.045) was more than twice as high and long-term use of benzodiazepines (2.0% vs. 1.0%, *p* = 0.059) was halved in 2008–11. Long-term use of synthetical anti-depressants (3.1% vs. 5.9%, *p* = 0.002) increased significantly while long-term use of phytoceutical anti-depressants (St. John’s wort) decreased significantly (2.0% vs. 0.5%, *p* = <0.001) (Fig. 1).

### Changes in psychotropic substance use according to socio-economic and health-related factors

**Fig. 2:**
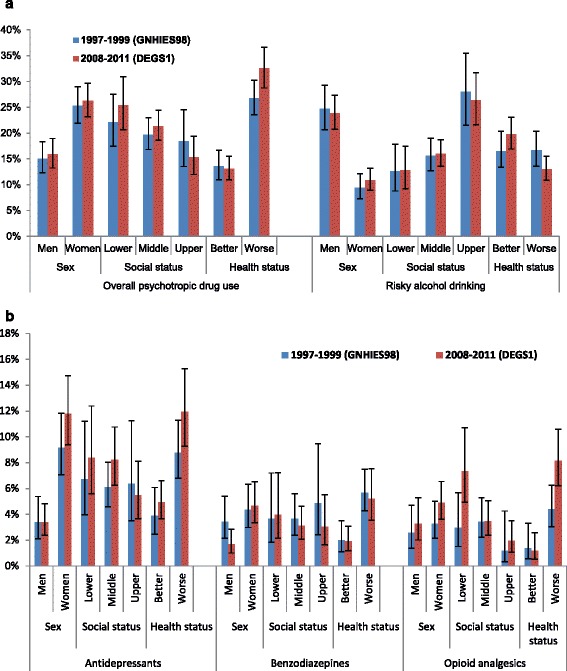
**a**. Overall psychotropic drug use and risky drinking among adults aged 60–79 years in Germany by sex, social status and self-assessed health status. National Health Interview and Examination Surveys GNHIES98 (1997-99) and DEGS1 (2008-11). Risky drinking: Average daily consumption of alcohol ≥10 g for women, and ≥20 g for men. **b**: Use of antidepressants, benzodiazepines and opioid analgesics among adults aged 60–79 years in Germany by sex, social status and self-assessed health status. National Health Interview and Examination Surveys GNHIES98 (1997–99) and DEGS1 (2008–11). Antidepressants: Synthetics (N06A) and St. John’s wort (N06AP01). Benzodiazepines: N05BA, N05CD, N03AE01 and N05CF. Opioid analgesics: N02A

Psychotropic drug use and risky alcohol consumption among elderly participants of the surveys of 1997–99 and 2008–11 differentiated according to sex, social status and self-assessed health status are presented in Figs. 2a and b. Among people with a worse health status overall psychotropic drug use increased from 26.8% to 32.5% (Fig. 2a) (*p* = 0.024) and the use of opioid analgesics rose from 4.4% to 8.1% (Fig. 2b) (*p* = 0.004). Further, among people with a lower social status the use of opioid analgesics increased from 2.9% to 7.3% (Fig. 2b) (*p* = 0.018) and among men the use of benzodiazepines decreased from 3.3% to 1.4% (Fig. 2b) (*p* = 0.023).

Figure 2a and b show that for the use of antidepressants, benzodiazepines and opioids gender gaps were widening and narrowing for risky drinking. Gaps between lower and upper social status were widening concerning the use of all groups of psychotropic drugs and narrowing for risky drinking. Gaps were also widening between those with a better and a worse health status for the use of overall psychotropic drugs, antidepressants, and opioids. Between those with a better and a worse health status gaps were also widening concerning risky drinking. This was due to fewer people with a worse health status and more people with a better health status engaging in risky drinking.

## Discussion

### Main findings

Overall psychotropic drug use remained stable while daily and moderate alcohol drinking increased between the two surveys of 1997-99 and 2008-11. Changes were observed for specific psychotropic drug groups and subgroups. Significant increases were found in the use of synthetical antidepressants (particularly SSRIs), Z-drugs, overall anti-dementia drugs (especially Ginkgo biloba), opioid analgesics, and anti-epileptics. The use of the phytoceutical antidepressant St. John’s wort and of benzodiazepines declined significantly. Significant rises were observed in the long-term use of opioid analgesics and synthetical anti-depressants, while the long-term use of benzodiazepines and St. John’s wort decreased significantly. Among people with a worse health status the use of overall psychotropic drugs and opioid analgesics increased significantly. Among those with a lower social status the use of opioid analgesics increased significantly while among men the use of benzodiazepines decreased significantly.

### Comparison to other studies

A comparison of prevalence rates has been described in our previous study [11]. In the present work we compared changes over time and direction of changes and found predominantly similar tendencies in psychotropic drug use in western countries. Prevalence rates of psychotropic drug use might vary between studies due to differences in observation periods, years of study conduction, settings, age groups, and drugs included. Differences in psychotropic drug use between countries could be due to differences in health care- and reimbursement systems, in understanding of psychiatric problems, cultural attitudes or traditions (i.e. Germany has a long tradition of phytoceutical therapies) [8, 23].

### Changes in overall psychotropic drug use and alcohol consumption

In our study overall psychotropic drug use remained unchanged between the two surveys of 1997-99 and 2008-11. A Finnish study [24] also reported unchanged prevalences of overall psychotropic drug use between 1990-91 and 1998-99 and a Swedish study [25] observed unchanged prevalences among cognitive intact elderly, but significant rises among cognitive impaired elderly between 1987 and 2007. Contrarily, significant increases in overall psychotropic drug use were reported by an US [26] and a Spanish study [27]. However, the US-study included only a three year observation period (1999–2002) which might cover a period with a higher increase and the Spanish study (1993 and 2003) involved older age groups (65+). Further, comparisons between countries might be difficult for the above described reasons.

In our study both, daily and moderate alcohol drinking increased significantly. Other studies described rises in risky drinking among the elderly [28, 29]. As in our analyses, higher prevalence rates of alcohol consumption were found among men [28–30] and narrowing gender gaps in the US, Australia, and European countries were described by a literature review of Keyes et al. [30] and a Swedish study [31]. Consistent with our findings, risky drinking was associated with a higher social status in some studies [32, 33]. In contrast to our results, risky drinking was associated with lower social class in an inner city study in the UK [34].

The growing alcohol consume among older people could be due to more healthy life years and improved incomes of the elderly. The rising alcohol consume in women could be due to a greater social acceptance and more financial independence [35]. Further, cohort effects and the impact of socio-historical contexts could influence drinking behavior [29].

### Changes in prevalence of specific psychotropic drug groups and subgroups

The significant increase in the use of synthetical antidepressants among the elderly, particularly of SSRIs, is also confirmed by other population based studies [24–26, 36, 37] as well as by studies based on dispensing-data [38, 39]. In our analysis the use of phytoceutical antidepressants (St. John’s wort) decreased significantly but international literature on changes/trends in the use of St. John’s wort is scarce. An American study on changes in sales of supplements [40] reported a peak in sales of St. John’s wort in 1998 followed by a decline until 2004 and then followed by stable sales until 2006. Further, similar as our study, other studies in Europe and the US observed a significant rise in the use of opioid analgesics [41–43], and Z-drugs [44] and a decrease in the use of benzodiazepines [44–46] among older people. In the present analyses we observed an increased use of overall anti-dementia drugs. This tendency was confirmed by a population-based Spanish study [47] and a Swedish study among residents of geriatric care [48]. The significant increase in the use of phytoceutical anti-dementia (Ginkgo biloba) in our study was also reported by an American study (1994–1999) [49], while a later conducted American study (1998–2002) [50] found a decrease in the use of Ginkgo biloba. In line with our study too, significant increases in the use of anti-epileptics were described by a Danish [51] and a Canadian [52] study as well as by a study comparing health record data of Spain, Denmark, Netherlands, the UK and Germany [53].

Rises in the use of synthetic antidepressants, anti-dementia, opioid analgesics and anti-epileptics might reflect a more efficient diagnosing, greater choice of medicines available, improved medical care for the elderly and improved adherence to guidelines. A German adaptation of the guidelines on Potential Inadequate Medication (PIM) for the elderly [54, 55] recommends for example to prescribe specific SSRIs (and other medicines) instead of a number of classical antidepressants of the ATC class N06AA, which could explain the significant rise in SSRI use among the elderly. The reasons for the decrease in the use of phytoceutical antidepressants (St. John’s wort) and the shift towards new synthetical antidepressants are unclear as studies, e.g. a meta-analysis of Cui (2016) [56] came to the result that for the treatment of mild and moderate depression St. John’s wort is equally effective as SSRIs and has fewer side-effects. The decrease in benzodiazepine use and a shift towards using more Z-drugs might reflect a growing concern about the side effects of benzodiazepines and a greater adherence to guidelines advising to prescribed Z-drugs instead of long-lasting benzodiazepines to older adults [54, 55]. Further, the guidelines recommend that older people use valerian (and other substances) instead of medium- and short-lasting benzodiazepines [54, 55]. However, a decrease in the use of valerian was found in our study. The increased use of opioid analgesics among older adults might be beneficial for pain reduction and improvement of physical function, but can also impair mental health functioning [57] and lead to falls and fractures [58].

### Changes in long-term-use of psychotropic drugs

As in our study, increases in long-term antidepressants use were also found for example in Finland [24] and the US [59]. Also comparable to our study, the long-term use of benzodiazepines decreased in a Finnish study [24]. A Dutch study [60] reported a decline in moderate duration of benzodiazepine use. Moderate duration of use was defined as ≥1 month – ≤1 year while long-term use was defined as ≥3 months in our study. Similar to other studies [43, 61, 62] we observed a significant rise in the long-term use of opioid analgesics among the elderly.

The increase in long-term use of synthetical antidepressants could be due to a better tolerability of new substances and a rise of treatment in accordance with medical guidelines. Increased use and long-term use of opioid analgesics could be due to changes in drug politics and due to rising diagnosis of pain [61, 62]. It might reflect a more adequate treatment of pain among older adults, although benefits and risks of long-term opioid use are discussed controversial in literature [4, 6, 43, 57].

### Changes in psychotropic drug use according to socio-demographic and health-related factors

In our analyses people with a lower social status were more likely to use opioids and those with a worse health status more likely to use overall psychotropic drugs and opioids, with gaps widening compared to their counterparts. A study comparing data from Canada, Albania, Colombia and Brazil [63] found a higher psychotropic drug use among people with a lower social status in Canada but not in Latin America. In Albania a lower social status was a predictor for higher use of anxiolytics, sedatives and hypnotics while antidepressants use was generally low [63]. In a Scottish study [41] use of strong opioids (classified according to the British National Formulary 2012) increased significantly and was associated with a lower social status and polypharmacy. In a Dutch study [60] benzodiazepine use remained stable between 1992 and 2002 and was associated with female sex, lower social status and a worse health status. Significant increases of psychotropic drug use among people with a worse health status were also reported by a Spanish study [36], while a Brazilian study [64] observed a significant rise in the use of antidepressants, which was associated with increased income and reasonable self-assed health status. Further, we found a decreasing benzodiazepine use in men which was confirmed by a Dutch study [60]. Our analyses showed a higher prevalence of psychotropic drug use in women and a growing gender gap over time which was also reported by other studies [24, 38, 43].

Differences in psychotropic drug use according to social status reflect the general social inequality of health and a higher use of psychotropic drugs among people with a worse health status was expectable. Reasons for older women’s higher consumption of psychotropic drugs might be rooted in different perceptions of mental or physical health [65], different patterns concerning the use of health-services [66], health professionals being more likely to prescribe psychotropic drugs to women [65], more inappropriate prescribing for women [66, 67] or differences in symptom patterns leading to underdiagnosing in men [68].

### Strengths and limitations

One of the strengths of our study is the large sample size of nation-wide and population based representative data. This allows generalization to the community dwelling population of Germany. Recall-bias concerning psychotropic drug use was minimized by limiting the observation window to 7 days prior to interview and by asking participants to bring along original packages of their medicines. Furthermore, recording all consumed medicines including privately prescribed, phytoceutical and OTC products, allowed us to analyse the “real” drug use among the community dwelling population. This is particularly relevant for the use of benzodiazepines as in Germany about 50% of benzodiazepine prescriptions are prescribed privately [7] and therefore are not recorded by health insurance data.

Limitations of our study include a selection bias as institutionalized people were excluded. Also, people with severe illnesses or cognitive impairments are underrepresented as they might not be able to come to the study centers. Further, the age limit of 79 years excludes those who might be mostly affected by medication use. An underestimation of psychotropic drug use among the elderly is therefore likely. It is also likely that we underestimated risky drinking as older people with severe alcohol problems might not come to examination sites.

## Conclusion

Psychotropic drug use among the elderly remained stable at a high level in Germany. Changes in the use of some psychotropic drug subgroups as well as of alcohol consumption patterns have been observed with significant rises in the use of synthetical antidepressants, opioid analgesics, Z-drugs, anti-dementia drugs, anti-epileptics, daily and moderate alcohol consumption. Female sex, lower social status and a worse health status were associated with psychotropic drug use in both surveys and gaps were growing compared to their counterparts. Rising alcohol consumption and rises in the use of potentially addictive drugs among the elderly are developments which could point towards a growing public health problem. Further studies are needed to evaluate health outcomes following changes in psychotropic substance use among the elderly.

## Abbreviations

ATC code: Anatomical therapeutic chemical code
CAPI: Computer assisted personal interview
CI: Confidence interval
DEGS1: German health interview and examination survey for adults 2008-11, first wave
GNHIES98: German national health interview and examination survey 1997-99
NSMRI: Non-selective monoamine reuptake inhibitors
OTC: Over-the counter
PIM: Potential inadequate medication
RKI: Robert Koch Institute
SSRI: Selective serotonin reuptake inhibitors
Z-drugs: Benzodiazepine-related drugs
